# A facile green and one-pot synthesis of grape seed-derived carbon quantum dots as a fluorescence probe for Cu(ii) and ascorbic acid†

**DOI:** 10.1039/d1ra05656e

**Published:** 2021-10-20

**Authors:** Jiawei Li, Ouwen Xu, Xiashi Zhu

**Affiliations:** School of Chemistry & Chemical Engineering, College of Guangling, Yangzhou University Yangzhou 225002 PR China zhuxiashi@sina.com

## Abstract

In this study, an on–off–on fluorescence probe for the detection of trace Cu(ii) and ascorbic acid (AA) based on biomass-derived sulfur and nitrogen double heteroatom-doped carbon dots (N,S-CDs) was designed. For the first time, the probe (N,S-CDs) was prepared from grape seeds and thiourea as the precursor. Cu(ii) was added to the carbon point solution, the fluorescence intensity (FL) of N,S-CDs was strongly quenched (switch OFF) and the fluorescence probe turned to “ON” (switch ON) with the addition of AA. Under the optimal conditions, the as-synthesized N,S-CDs had a good detection performance for Cu(ii) and AA assay with the linearity ranges from 150–500 μg mL^−1^ and 0.1–400 μg mL^−1^, and the LODs were 0.048 mg L^−1^ and 0.036 mg L^−1^, respectively. The as-prepared N,S-CDs exhibited a low cytotoxicity and a good biocompatibility, which show their potential for application in the biological imaging of living cells.

## Abstract 2

In this study, an on–off–on fluorescence probe for the detection of trace Cu(ii) and ascorbic acid (AA) based on biomass-derived sulfur and nitrogen double heteroatom-doped carbon dots (N,S-CDs) was designed.

## Graphical abstract

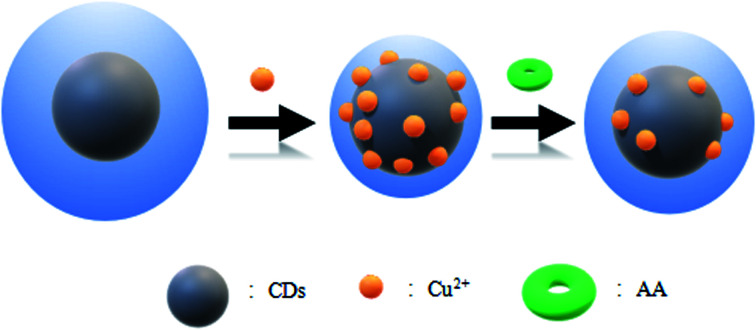

## 1. Introduction

Carbon dots (CDs) have been applied in pharmaceutical analysis, bio-imaging and other fields due to their advantages of high photostability, good biocompatibility, low toxicity, and excellent optical properties.^1^ The carbon source is one of the important conditions that directly affects the properties of the CDs.^2^ Carbon sources mainly include organic chemicals (citric acid,^3^*etc.*) and biomass (plants,^4^ animals^5^ and their derivatives^6^). Due to the high nitrogen content,^7^ low cost, and good biocompatibility of biomass,^8^ more and more biomass-derived carbon dots were synthesized, such as soy milk^9^ (Yu = 8.6%, Yu is the fluorescence quantum yield) and bamboo leaves^10^ (Yu = 7.1%). Heteroatom doping is a conventional technique to modify CDs.^11^ Nitrogen,^12^ phosphorus,^13^ boron^14^ and sulfur^15^ are often doped into CDs. Heteroatoms can be introduced to the surface of CDs to provide more active centers, and add the carbon core of CDs to adjust the bandgap and establish new energy levels,^16,17^ thereby greatly enhancing the fluorescence intensity of doped CDs.

Grape is one of the widely grown fruit crops in the world, with an annual output of about one million ton. Every year, a large amount of grape waste (peels and seeds) is produced, accounting for 13.5–14.5% of the total production.^18^ This type of biological residue is usually used as a fertilizer or discarded. However, its seeds contain numerous polyphenols,^19^ which can be used as a good carbon and nitrogen source for CD production. In this study, grape seed was used as the carbon source, thiourea was used as the source of doping elements, and nitrogen and sulfur introduction was carried out for the first time for the facile one-pot synthesis of biomass-derived N–S-CDs. The fluorescence probe was designed by quenching N,S-CDs with Cu(ii), followed by a fluorescence recovery by the reaction of Cu(ii) with AA. The fluorescence changes of N,S-CDs were directly related to the concentrations of AA and Cu. Further, the fluorescence probe had advantages in the detection of Cu(ii) and AA in real samples, and showed a good result. Besides, N,S-CDs have been used in cells DU145 for cell imaging.

## 2. Experimental

### 2.1. Chemicals and materials

Grape seeds were purchased from Chinese pharmacy (Yangzhou, China). Quinine sulfate was purchased from Aladdin (Shanghai, China). CuSO_4_, FeCl_3_·6H_2_O, FeSO_4_, CoCl_2_, AgNO_3_, Pb(NO_3_)_2_, ZnSO_4_, Cr(NO_3_)_3_, NiSO_4_, MgCl_2_, CaCl_2_, MnCl_2_, AlCl_3_, NaCl and CH_4_N_2_S were purchased from Sinopharm Chemical Reagent Co, Ltd (Shanghai, China). All reagents were of analytical reagent grade.

### 2.2. Instruments

A BSA224S Analytical balance (Suzhou Sainz Instrument Co., Ltd), MARS 6240/50 Microwave Digestion Apparatus (Peian Technology Company, USA), PHS-25 pH meter (Shanghai INESA Electronics Co, Ltd), F-7000 Fluorescence spectrophotometer (Hitachi, Japan), KQ-50E Ultrasonic cleaner (Kunshan Ultrasonic Instrument Co. Ltd), and thermo-1780 Ultraviolet spectrophotometer (Thermo Fisher Scientific, Inc.) were used to carry out the experiment.

### 2.3. Synthesis of N,S-CDs

We washed grape seeds and dried them in a drying box. Then, the grape seeds were ground into powder and used as the carbon source for synthesising N,S-CDs. 2.50 g of this powder and 3.0 g of thiourea were mixed into a 25.0 mL aqueous solution under magnetic stirring for 30 min. The solution was transferred to a polytetrafluoroethylene autoclave and heated at 200 °C for 8 h. After cooling down to room temperature, the resultant dark brownish suspension was centrifuged at 10 000 rpm for 20 min to remove large dots. The transparent solution was dialyzed for 24 h using a dialysis membrane (cut off 1/4 3500 Da).

### 2.4. Fluorescence quantum yield (Yu)

We measured quantum yield (Yu) *via* the reference method.^20^ Under the same wavelength, the absorbance, FL and integral FL of the two diluted solutions of the test sample and the reference substance were respectively measured, and calculated by substituting them into the following eqn (1)

1 Yu = Ys × Fu/Fs × As/Au

Yu is the fluorescence quantum yield of N,S-CDs, Ys is the fluorescence quantum yield of the reference standard substance (quinine sulfate), Fu is the integrated FL of N,S-CDs, Fs is the integrated fluorescence intensity of quinine sulfate, Au is the absorbance of the N,S-CDs, and As is the absorbance of quinine sulfate at the excitation wavelength.

### 2.5. Recommended procedure

For the determination of Cu(ii) and AA, 0.50 mg mL^−1^ of N,S-CD aqueous solution (1.0 mL) was mixed with a buffer solution (ammonium acetate buffer, pH = 7) and the undetected solution to obtain a 4 mL solution. The fluorescence emission spectra of Cu(ii) and AA in a solution were recorded at an excitation wavelength of 430 nm and emission wavelength of 490 nm, and both excitation and emission slits were 5.0 nm.

### 2.6. Preparation of real sample

Water samples were pre-treated with a 0.22 μm micro-pore film filter. The actual water samples were added with different concentrations of the Cu(ii) standard solutions and analyzed without any pretreatment.

For the fruit juice, different concentrations of AA were introduced into 0.5 mL fruit juice. After that, the mixture was centrifuged at 80 000 rpm for 30 min. Finally, the sample was collected and used for the detection of AA.

### 2.7. Cell viability assay

MTT assay was used to determine the cell viability. The cells were incubated with different concentrations of N,S-CDs for 24 h, with cells treated with 0.1% DMSO as the control. After 24 h of incubation, the cells were added with 20 μL 5 mg mL^−1^ MTT of glucose, and then cultured at 37 °C for a 4 h incubation. The optical density (OD) at 470 nm of the final nail was measured on a universal microplate reader EL800. The cell proliferation inhibition rate was calculated according to the formula: 1 − OD (experiment)/OD (control) × 100%.

### 2.8. Cellular imaging

Human prostate epithelial cell line RWPE-1 and human prostate cancer cell line DU145 were obtained from the cell bank of Shanghai Institute of Biochemistry and cell biology, Chinese Academy of Sciences. RWPE-1 cells were grown in DU145 cells in RPMI-1640 supplemented with 10% fetal bovine serum (Gibco, Waltham, USA), 100 U mL^−1^ penicillin, and 100 U mL^−1^ streptomycin, which was cultured in a humidified incubator (5% CO_2_, 37 °C).

## 3. Results and discussion

### 3.1. Characterization of N,S-CDs

The morphology, structure, elements, and surface groups of the N,S-CDs were analysed.

**Fig. 1:**
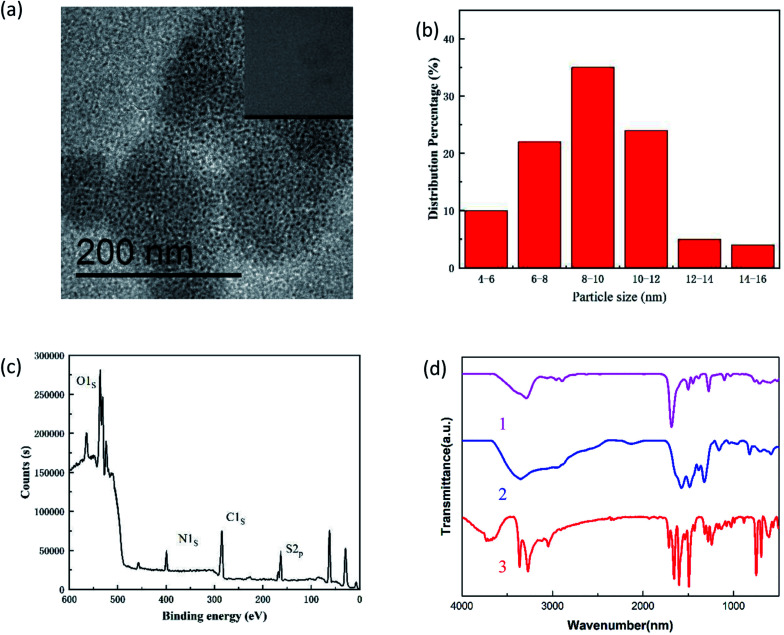
Characterization of the N,S-CDs. (a) TEM image of N,S-CDs and HRTEM image of N,S-CDs (inset view); (b) the size distribution of the N,S-CDs; (c)XPS spectra of N,S-CDs; (d) FT-IR spectra of grape seeds (curve 1), thiourea (curve 2) and N,S-CDs (curve 3).

The TEM image of newly prepared N,S-CDs is shown in Fig. 1a. The well-monodispersed N,S-CDs had a uniformly spherical shape, with an average diameter of 8.9 nm and a concentrated size distribution (Fig. 1b). In addition, the HRTEM image (inserted in Fig. 1a) reveals a clear crystal lattice distance of 0.24 nm, similar to the (1120) lattice fringe of graphene. The above observations indicated that N,S-CDs had crystalline properties, as evidenced by a clear fringe distance of 0.22 nm, which was similar to the (1120) characteristic lattice fringes of graphene.^21^ The analysis results of XPS are shown in Fig. 1c. Four peaks around 165.18 eV, 285.12 eV, 532.12 eV, and 400.12 eV can be attributed to sulfur, carbon, oxygen and nitrogen, respectively, indicating the successful doping of N and S atoms. FT-IR spectroscopy was used for the identification of the functional groups in N,S-CDs. As shown in Fig. 1d, curve 1, curve 2 and curve 3 were the FT-IR spectra of grape seed, thiourea and N,S-CDs, respectively. As for curve 1, the peak at 3510 cm^−1^ was induced due to the O–H or N–H stretching vibrations, whereas the peaks at 3040 and 1595 cm^−1^ are attributed to the C–H and C=O stretching vibrations, respectively. The absorption at 1451 cm^−1^ was attributed to the C–N stretching vibration, and compared to curve 2, the new absorption at 1123 cm^−1^ was attributed to the stretching vibrations of C–S. The FTIR and XPS results demonstrated that the as-synthesized N,S-CDs had different chemical bonds and surface functional groups, which indicated that the N,S-CDs had good water solubility, and the N,S-CDs were synthesized successfully.

### 3.2. Properties of N,S-CDs

#### 3.2.1. Basic properties of N,S-CDs

**Fig. 2:**
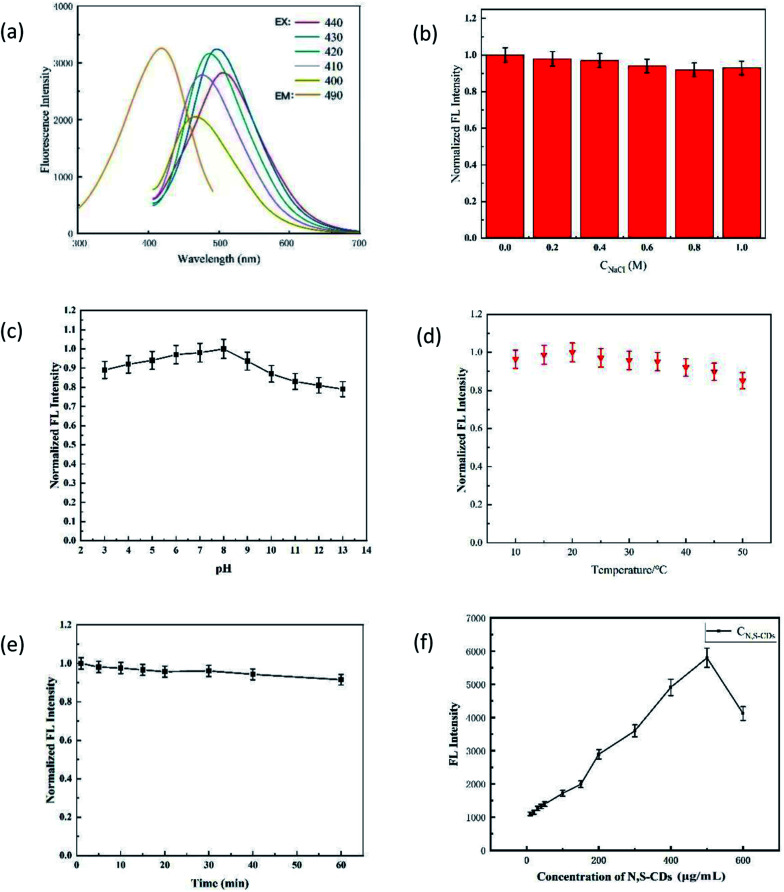
Basic properties of N,S-CDs. (a) Fluorescence excitation spectra and emission spectra of N,S-CDs. Normalized FL intensity of N,S-CDs (b) in the presence of various concentrations of NaCl; (c) at different pH; (d) at different temperatures; (e) irradiated with a Xe lamp for 60 min; (f) optimization of the concentration of N,S-CDs.

**Fig. 3:**
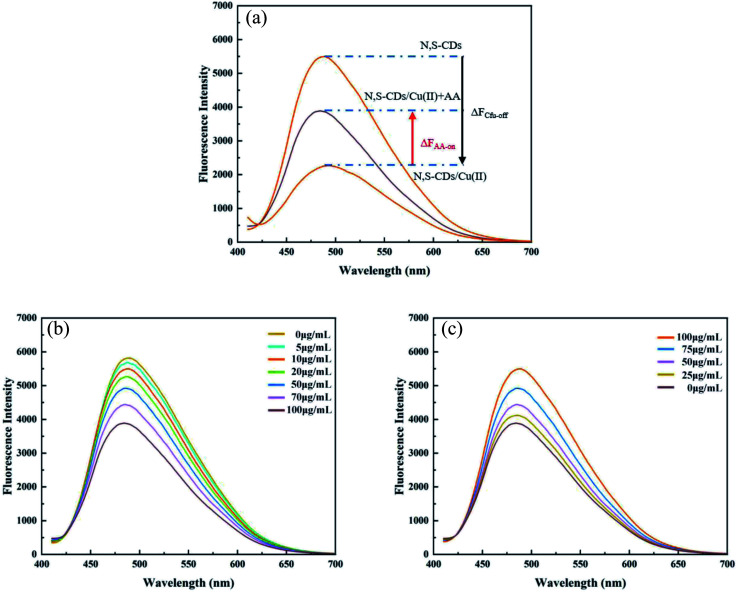
Fluorescent emission spectra. (a) Fluorescent emission spectra of the N-CD aqueous solutions upon the addition of Cu(ii) and FL spectra of the N-CDs/Cu(ii) system upon the subsequent addition of AA. (b) Fluorescence spectra after adding Cu(ii) concentration of 0–100 μg mL; (c) fluorescence spectra after adding AA concentration of 0–100 μg mL^−1^.

**Fig. 4:**
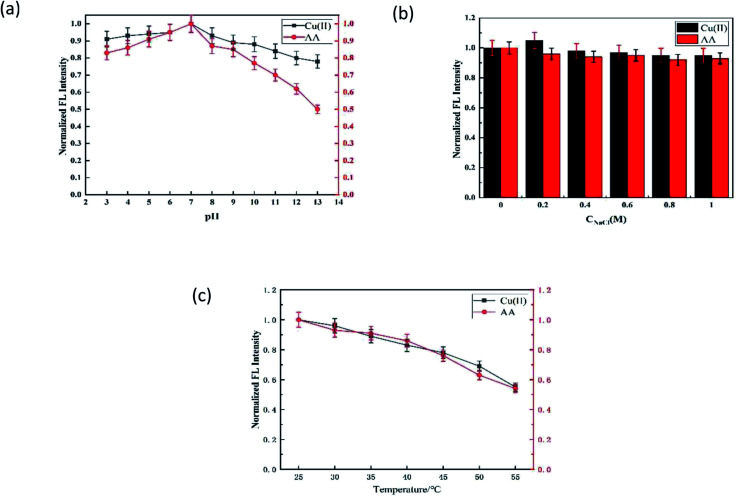
Optimization of the detection conditions for Cu(ii) and AA. Effect of (a) pH, (b) ionic strength (c) and temperature.

**Fig. 5:**
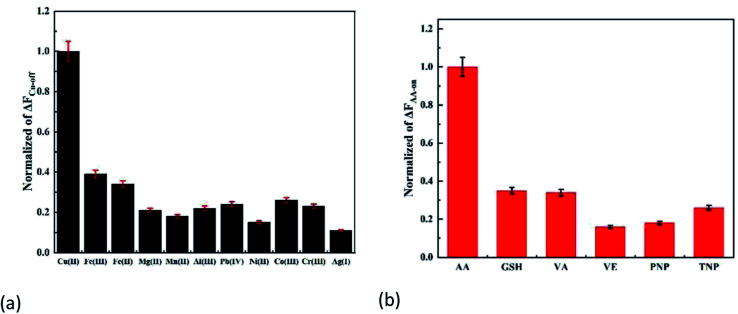
Selectivity of (a) Cu(ii) and (b) AA.

Fig. 2 shows the basic properties of N,S-CDs. It can be seen that: (1) the fluorescence spectra of N,S-CDs are excitation-dependent with the increase in the excitation wavelength from 400 to 440 nm (Fig. 2a); (2) N,S-CDs had a good salt tolerance (the fluorescence intensity (FL) did not fluctuate even when the concentration of ions reached up to 1.0 M) (Fig. 2b); (3) *F* was stable in the pH range of 3.0–6.0 (Fig. 2c) in the temperature range of 15–40 °C (Fig. 2d); (4) *F* decreased slightly with the increase in the irradiation time (Fig. 2e); (5) the fluorescence self-quenching would occur when N,S-CDs concentration exceeded 500 μg mL^−1^.

#### 3.2.2 “On–off–on” property of N,S-CDs

N,S-CDs exhibited the properties of a switched fluorescent probe (ON–OFF–ON). Cu(ii) could “turn off” the signal of N,S-CDs (Δ*F*_Cu-off_) and AA could “turn on” the signal of N,S-CDs/Cu (Δ*F*_AA-on_) (Fig. 3a). The concentrations of Cu and AA were linearly related to Δ*F*_Cu-off_ and Δ*F*_AA-on_ within a certain range (Fig. 3b and c). Therefore, N,S-CDs can be used as the probe for the detection of Cu(ii) and AA.

### 3.3. Optimization of the detection conditions for Cu(ii) and AA

In this experiment, the pH value, ionic strength, and reaction temperature were optimized.

The influence of pH on the fluorescence probe is shown in Fig. 4a. Δ*F*_Cu-off_ and Δ*F*_AA-on_ had similar changing trends with pH, and both had the best values when pH = 7.0. The reasons for this phenomenon may be the same as that Δ*F*_Cu-off_ and Δ*F*_AA-on_ that (1) the interaction between N,S-CDs and Cu(ii) would be enhanced with the increase in pH (3.0–7.0) (Δ*F*_Cu-off_↑), and weakened with the increase in pH (7.0–13.0) due to the side reaction coefficient of hydroxyl increased in the alkaline medium (Δ*F*_Cu-off_↓);^22–24^ (2) AA in the enediol group and the lactone group were easily deprotonated to radical cation at suitable pH, thus binding with Cu(ii) stably (Δ*F*_AA-on_↑); however, the hydrolysis of the lactone ring in AA and the structure of AA would be destroyed under alkaline conditions, and the hydrolysis of AA was greater than the redox effects.^25^ Therefore, the pH value of the system was controlled to 7.0 in the process of detecting Cu(ii) and AA.

The effect of the ionic strength on the results of determination can be ignored (Fig. 4b). The Δ*F*_Cu-off_ and Δ*F*_AA-on_ reached the maximum at 30 °C, which then decreased with the increase in the temperature (Fig. 4c).^26^ Thus, the optimized pH of 7.0 and temperature of 25 °C were adopted in the following experiments.

### 3.4. Selectivity of Cu(ii) and AA

Different interferences were selected to evaluate the potential selectivity of the method. As shown in Fig. 5a and b, Δ*F*_Cu-off_ and Δ*F*_AA-on_ did not change obviously in the presence of the same concentration of interferences, and the corresponding effect was the best. The results indicated that the interferences can be ignored.

The allowable interference multiple (±5%) of Cu(ii) and AA detection were determined (Table 1). It can be shown that the probe had a strong anti-interference ability.

**Table 1:** Anti-interference capability (±5%)

Interference of Cu	Multiple (M/Cu(ii))	Interference of AA	Multiple (M/AA)
Fe(iii)	3	GSH	2
Fe(ii)	15	VE	3
Mg(ii)	50	VA	5
Co(ii)	50	PNP	10
Ca(ii)	50	TNP	10
Ni(ii)	100	—	—
K(i)	100	—	—

### 3.5. Analysis performance of Cu(ii) and AA

The specifically analyzed performances are shown in Table 2. Compared to other detection methods, the LODs of Cu(ii) and AA were much lower than those of in previous studies(Table 3).

**Table 2:** Analysis performance of Cu(ii) and AA

	Cu(ii)	AA
Linear	*y* = 7.3180 × 10^−4^*x* + 0.0626	*y* = 0.0059*x* + 1.084
*R* ^2^	0.99908	0.99102
Linear range	0.150–500 μg mL^−1^	0.100–400 μg mL^−1^
LOD	0.048 μg mL^−1^	0.036 μg mL^−1^
RSD	0.28%	0.41%

**Table 3:** Comparison of detection performance of different fluorescent probes for Cu^2+^ and AA detection

Raw material	Synthetic method	Yu (%)	LOD of Cu(ii) μg mL^−1^	LOD of AA μg mL^−1^	Reference
Mint	Microwave	17	—	0.053	6
Flax straw	Hydrothermal	20.7%	—	0.158	32
Hemicelluloses	Hydrothermal	16.6%	0.054	—	33
Bamboo leaves	Hydrothermal	7.1	0.064	—	34
Grape seeds	Hydrothermal	27.5%	0.048	0.036	This work

### 3.6. Detection of Cu(ii) and AA in real samples

Tap water, Baodai River, lemon juice, and orange juice were selected as real samples to explore the practicability of N,S-CDs in the detection of real samples. The results are shown in Tables 4 and 5. No copper ion was detected in real samples. The concentration of AA in the lemon juice and orange juice were 198 μg mL^−1^ and 43.2 μg mL^−1^, respectively. The recoveries of Cu(ii) and AA in these samples were 97.5–99.4% and 98.1–102.0%, respectively. These results show that N,S-CDs can be used to detect Cu(ii) and AA in practical samples with excellent performances.

**Table 4:** Determination of Cu(ii) in real water samples

Sample	Cu(ii) added (μg mL^−1^)	Cu(ii) found (μg mL^−1^)	Recovery (%)
Tap water	0	—	—
	2.00	2.09	104.50%
	4.00	3.83	95.75%
	6.00	6.14	102.33%
	8.00	7.93	99.13%
Baodai river	0	—	—
	2.00	1.96	98.00%
	4.00	4.13	103.25%
	6.00	6.09	101.50%
	8.00	7.86	98.25%

**Table 5:** Determination of AA in fruit juices

Sample	Added (μg mL^−1^)	AA found (μg mL^−1^)	Recovery (%)
Lemon juice	0.00	201.1	—
	11.7	208.9	98.1
	23.4	223.0	99.4
	35.1	234.0	99.1
	46.8	246.6	97.5
Orange juice	0.00	43.2	—
	11.7	56.1	102.2
	23.4	65.3	98.1
	35.1	77.3	98.7
	46.8	92.3	102.5

### 3.7. Fluorescence quenching mechanism

There are numerous reasons for fluorescence quenching, such as electron transfer, molecular rearrangement, formation of nonfluorescent complexes and intermolecular collision quenching,^27^ which can be discussed by quenching kinetics, UV spectrum and zeta potential.

#### 3.7.1. Quenching kinetics

**Fig. 6:**
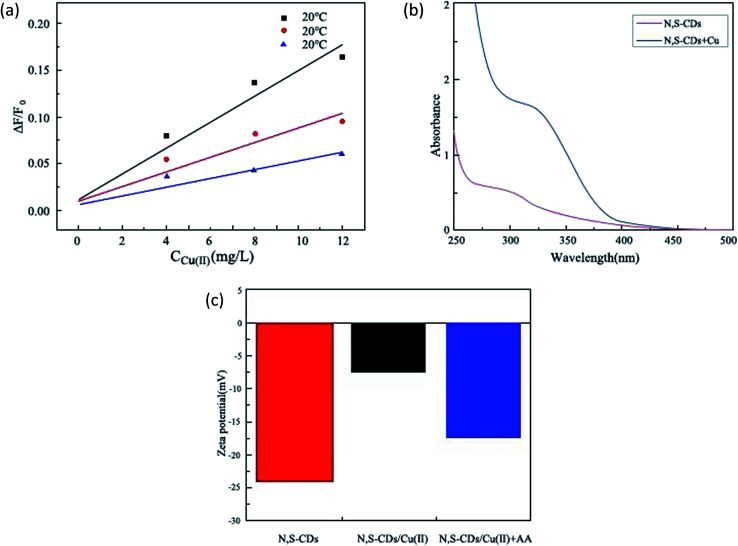
Mechanism of fluorescence “off”. (a) Quenching Stern–Volmer plot of N,S-CDs in the presence of different concentrations of Cu(ii) at different temperatures; (b) UV-Vis absorption spectra of N,S-CDs (1), N,S-CDs/Cu(ii) (2); (c) zeta potentials of N,S-CDs, N,S-CDs/Cu(ii) and N,S-CDs/Cu(ii) + AA.

Quenching mechanisms can be divided into two categories: dynamic quenching and static quenching.^27^ It is a common method to judge a quenching type by Stern–Volmer quenching constant KSV. The Stern–Volmer parameters of 20 °C, 40 °C, and 60 °C were measured and computed. It could be clearly seen from Fig. 6a that the KSV value decreased with the increase in temperature, which was consistent with the characteristics of static quenching, therefore, it can be initially judged that the process of Cu(ii) quenching of N,S-CD fluorescence was a static quenching process. A non-fluorescent matrix complex is formed by the interaction between the acceptor molecule [Cu(ii)] and fluorescent molecules (N-CDs), in which the intermolecular interaction forces included hydrogen bonding, hydrophobic, electrostatic and van der Waals force interactions. Through further analysis of the fitting results in Table S1,† the thermodynamic parameters of the reaction Δ*H*^Θ^ (enthalpy change) and Δ*S*^Θ^ (entropy change) can be obtained, which can be investigated during the sudden extinction process of molecular interaction forces. The formula is as follows:ln *K* = −(Δ*H*^Θ^/*RT*) + (Δ*S*^Θ^/*R*)where *K* is the temperature quenching slope, *T* is the corresponding temperature, and *R* is the gas constant. The calculation results are shown in Table S2.† When Δ*H*^Θ^ < 0 and Δ*S*^Θ^ > 0, the reaction proceeded spontaneously. According to the rule summarized by Ross and Subramanyam, the static burst process between N,S-CDs and Cu(ii) at Δ*H*^Θ^ < 0 and Δ*S*^Θ^ > 0 was mainly caused by the charge interaction.^28^

#### 3.7.2. UV absorption spectrum

The UV-Vis absorption spectrum of the probe was investigated, which confirmed the fluorescence static quenching mechanism of the probe. As shown in Fig. 6b, the UV-Vis spectrum of N,S-CDs/Cu(ii) solution after fluorescence quenching was different from those of N,S-CDs and Cu(ii), explaining the binding interactions of N,S-CDs and Cu(ii).^29^ Since new absorption peaks appeared in the absorption spectrum before and after quenching, non-fluorescent ground state was produced.^31^

#### 3.7.3. The zeta potential

The zeta potential is shown in Fig. 6c. The zeta potential of N,S-CDs was −35.3 mV because there were many –COOH on the surface of N,S-CDs. In the presence of 40 μM Cu(ii), the zeta potential of N,S-CDs increased to −3.4 mV, which may be because when –COOH and Cu(ii) coordinate to form a ground state complex, oxygen will obtain a positive charge, leading to the increase in the zeta potential.^30^ Therefore, it once again proved that the fluorescence behaviour of the N,S-CDs/Cu(ii) system is a kind of static fluorescence quenching.

### 3.8. Fluorescence recovery mechanism

The restore of fluorescence in the system was mainly attributed to the competitive coordination mechanism and redox mechanism. For the competition mechanism, Cu(ii) could chelate with several hydroxyl groups in AA efficiently, taking Cu(ii) away from the N,S-CD/Cu-based non-luminescence and making the N,S-CD return to the original state, leading to the result that fluorescence reappeared and “turned on”. Simultaneously, some researchers believe that Cu(ii) can be reduced to Cu(i) by AA, and Cu(i) has no empty orbit for –COOH and –NH_2_ of the N,S-CDs, which makes Cu(ii) separate from the surface of the N,S-CDs, and leads to the fluorescence recovery.^35^,^36^

### 3.9. Biological assay

**Fig. 7:**
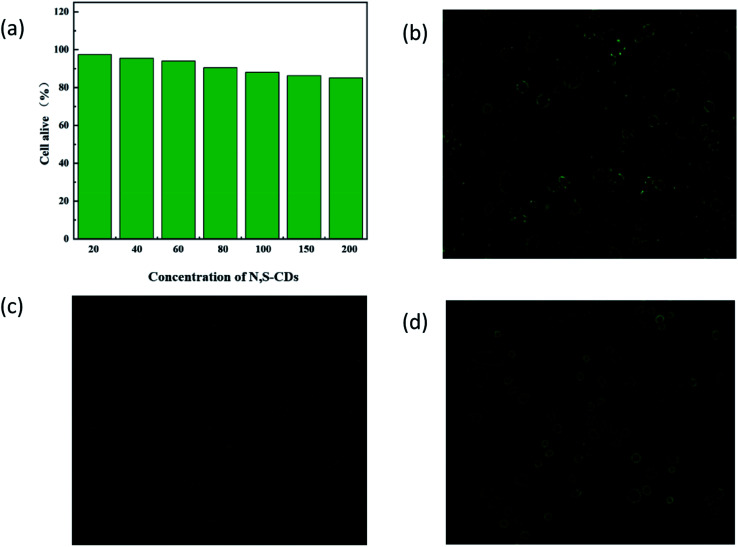
Cell Viability values and Cell imaging. (a) Viability values (%) of cells estimated by the MTT assay *versus* incubation concentrations of the N,S-CDs; (b) DU145 cells incubated with N,S-CDs (green channel); (c) DU145 cells incubated with N,S-CDs/Cu(ii) (green channel); (d) DU145 cells.

It is necessary to investigate the biocompatibility of N,S-CDs before cell imaging. Therefore, the cytotoxicity of N,S-CD was studied using human DU145 cells in the MTT assay. The results are shown in Fig. 7a. Evidently, after 24 h of incubation with 200 mg mL^−1^ N,S-CDs, the cell viability still exceeded 85%. Therefore, the as-synthesized N,S-CDs were reliable for cell imaging *in vitro*. According to Fig. 7b, DU145 cells were incubated in the N,S-CD solution (20.0 mg mL^−1^), and the green excitation image clearly showed a strong green fluorescence, indicating that a large number of N,S-CDs penetrated into the cell membrane, and no photoluminescence was observed in the nucleus. Subsequently, a 50.0 mM Cu(ii) solution was added to DU145 cells treated with N,S-CDs, and the intracellular fluorescence decreased significantly (Fig. 7c). When the cells incubated with N,S-CDs/Cu(ii) were further treated with an increased concentration of AA, the gradually restored fluorescence is observed in the cells, as shown in the Fig. 7d. These observations support a highly selective and switchable fluorescence process in living cells. Therefore, the probe has a potential application value in prostate cancer cell imaging.

## 4. Conclusion

This study attempted to develop an on–off–on probe based on a simple, economical and eco-friendly hydrothermal synthesis of N,S-CDs using grape seeds and thiourea as the starting materials. The results provided that the method can be used for the determination of Cu(ii) and AA in real samples, and had broad application prospects in water quality monitoring, food quality supervision, biological imaging and other fields.

## Conflicts of interest

There are no conflicts to declare.

## Supplementary Material

RA-011-D1RA05656E-s001
