# Supplementary material for: A facile green and one-pot synthesis of grape seed-derived carbon quantum dots as a fluorescence probe for Cu(ii) and ascorbic acid
Source: RSC Adv. 2021 Oct 20;11(54):34107–16. doi: 10.1039/d1ra05656e (PMC9042380; doi:10.1039/d1ra05656e)
Supplement: RA-011-D1RA05656E-s001 [file RA-011-D1RA05656E-s001.pdf]

Electronic Supplementary Material (ESI) for RSC Advances.
This journal is © The Royal Society of Chemistry 2021

Support materials

Facile green and one-pot synthesis of Grape seeds derived carbon quantum dot as a fluorescence probe for Cu(II) and ascorbic acid

Jiawei Li, Ouwen Xu and . Xiashi Zhu .

School of Chemistry & Chemical Engineering, College of Guangling, Yangzhou University, Yangzhou, 225002, PR China

*corresponding email address: xszhu@yzu.edu.cn; zhuxiashi@sina.com (X.S. Zhu)

Table S1. Fluorescence quenching constant of different temperature

T	20°C	40°C	60°C
K_{SV}	0.812	0.736	0.721

Table S1. Calculation results of Fluorescence quenching constant in different temperature

Table S2 Thermodynamic calculation

Temperature T/K	$\triangle H^{\Theta}/(J{\bullet}mol^{-1})$	$\triangle S^{\Theta}/(J{\bullet}mol^{-1}{\bullet}k^{-1})$
293.15	-1702.83	8.549

Table S2. Calculation results of Fluorescence quenching Thermodynamic calculation
